# 

**Published:** 1896-09

**Authors:** 


### CONTENTS.

ORIGINAL ARTICLES—

Some Obstetrical Cases. By W. M. Holladay, A. B., M. D., Hampden Sidney, Va.. .......... 427
The Placenta, How and When Delivered. By J. B. Murfree, M. D., Murfreesboro, Tenn.......... 432
Is the Diagnosis of Skin Diseases Necessary? By M. B. Hutchins, M. D.. .......... 438
Strangulated Hernia. By Virginius W. Harrison, A. M., M. D., Richmond, Va.......... 441

SELECTIONS AND ABSTRACTS—

How Shall We Feed the Baby? By John T. Winter, M. D., Washington, D. C.......... 450
Peroxide of Hydrogen.......... 457
Beri-Beri in the Fiji Islands.......... 460
Doctors Should not be so Modest.......... 461

SOCIETY REPORTS—

Richmond Academy of Medicine and Surgery .............. 463

EDITORIAL—

Announcement Extraordinary .............................. 468
Thyroid or Thyreoid ..................................... 468
A Successful Laparotomy at Eighty-one Years .............. 468

NOTES— 469

BOOK REVIEWS— 472

SPECIAL NOTES— 474

PRESCRIPTIONS— 478

